# Social enrichment mitigates facial expressions and physiological indicators of short-term stress in horses

**DOI:** 10.1371/journal.pone.0347571

**Published:** 2026-04-20

**Authors:** Ana Caroline Bini de Lima, Vanessa Cristini Sebastião da Fé, Maria Simara Palermo Hernandes, Emily Caroline Pfeifer de Cristo, Ana Gabrieli dos Santos Fagundes Euzébio, Maria Vitória e Silva Sousa, Fabiana Ribeiro Caldara, Viviane Maria Oliveira dos Santos

**Affiliations:** 1 Graduate Program in Animal Science, Faculty of Veterinary Medicine and Animal Science, Federal University of Mato Grosso do Sul, Campo Grande, Brazil; 2 Undergraduate Program in Animal Science, Faculty of Veterinary Medicine and Animal Science, Federal University of Mato Grosso do Sul, Campo Grande, Brazil; 3 Graduate Program in Animal Science, Faculty of Agricultural Sciences, Federal University of Grande Dourados, Dourados, Brazil; Universidade Federal de Mato Grosso do Sul, BRAZIL

## Abstract

This study aimed to evaluate the ability of social noncontact environmental enrichment to facilitate social buffering and to characterize the emotional experience of horses subjected to restraint in stock by assessing physiological parameters and facial expressions. Pantaneiro horses (n = 11) were evaluated in a crossover design with two treatments: social noncontact enrichment during stock restraint and social isolation during stock restraint. Physiological parameters (heart rate, heart rate variability, respiratory rate, ocular temperature by infrared thermography, and auricular temperature by infrared thermometer) and facial expressions (EquiFACS) were assessed throughout the 24-minute restraint period. When horses were accompanied by a conspecific, heart rate, respiratory rate, and eye temperature were lower (p < 0.05) than when they were socially isolated. The frequency of facial expressions associated with stress responses, such as *nostril dilator* (AD38), *inner brow raiser* (AU101), *upper eyelid raiser* (AU5), *eye white increase* (AD1), *ears forward* (EAD101), and *ears back* (EAD104), was also lower (p < 0.05) in social noncontact enrichment compared to social isolation. The combined assessment of facial expressions and physiological parameters provides robust evidence that, during this intervention, the animals experience an emotional state characterized by high arousal and negative valence. In this context, social noncontact environmental enrichment can facilitate social buffering, leading to a reduction in stress indicators associated with high arousal and negative valence.

## Introduction

Horses face challenges inherent to the domestic environment, as they are constantly subjected to procedures and management practices that can influence their emotional state by limiting the expression of natural species-specific behaviors. The use of restraint stocks, typically constructed from solid posts, is common in handling procedures to facilitate veterinary care, rectal palpation, ultrasonographic examination, and other reproductive and health-related procedures [1]. However, this type of intervention imposes considerable movement restriction, which, for a species whose primary defense strategy is flight, can be aversive even for habituated animals and may induce a short-term stress response [2,3].

Although animal welfare research has traditionally focused on reducing the occur-rence of aversive experiences, as they can lead to poor welfare conditions, some potentially stressful procedures are inherent to good husbandry practices [4]. This is the case with re-straint handling, which, despite being a challenging event for horses, facilitates animal care and health maintenance. In this context, it is necessary to seek alternatives that help horses cope with the challenges to which they are routinely exposed and promote stress reduction during interventions.

The introduction of a conspecific, that is, an individual of the same species, as a method of social environmental enrichment can be beneficial when the animal faces ad-versity, as social support has buffering effects that mitigate the negative impact of stress-ors on the individual’s welfare, a phenomenon known as social buffering [5,6]. This demonstrates that the presence of a compatible companion has the potential to attenuate the stress response and/or allow for faster recovery of the individual after a challenging event.

Social enrichment involves the socialization of animals with conspecifics or heter-ospecifics, both in conditions where direct contact is provided (social contact enrichment) and in conditions where no contact occurs (social noncontact enrichment), with interac-tions in the latter case being facilitated through visual, auditory, and olfactory communication [7,8]. Social noncontact enrichment can be an interesting strategy because, even though animals cannot engage in normal species-specific physical interactions, they can remain aware of the presence of conspecifics in the area and respond to the sensory cues provided by them. Thus, sensory cues can serve as an alternative to direct contact [9].

To assess whether the presence of a conspecific can promote social buffering, it is necessary to use stress-indicator parameters. The physiological changes that constitute the stress response can be objectively measured through non-invasive parameters such as respiratory rate (RR) [10,11], heart rate (HR), heart rate variability (HRV) [12,13], and ocular temperature by infrared thermography [14–16]. However, this assessment is more robust when a combination of physiological and behavioral measures is used [17]. In this regard, the evaluation of facial expressions deserves attention due to their potential to re-flect the animal’s internal state more reliably, compared to general behavior [18].

Facial Action Coding Systems (FACS) provide a systematic methodology for identi-fying and coding facial expressions based on underlying facial musculature and muscle movement [19,20]. Recently, a Facial Action Coding System (FACS) specifically designed for horses, known as EquiFACS, was developed. It provides a comprehensive list of all the facial movements that individuals of this species are capable of producing [19]. Moreover, this tool has been successfully employed in a recent study focused on identifying relevant facial expressions exhibited by horses during stressful interventions [21].

Facial expressions can also provide valuable insights into horses’ emotional experi-ences [18,22]. Through the dimensional approach, it is possible to characterize emotional experiences in terms of valence (positive/pleasant/attractive or negative/unpleasant/aversive) and arousal level (high or low) [23,24]. Evidence from equine research indicates that stressful situations generally lead to the production of facial expressions associated with high arousal and negative valence [21,25–27].

Our previous study demonstrated that sensory environmental enrichment, specifically auditory stimulation, can modulate both facial and physiological responses in horses subjected to short-term stressors, such as movement restriction and social isolation [27]. However, the characterization of the emotional experience of horses subjected to movement restriction due to stock handling, both with and without access to social environmental enrichment, remains to be tested.

This study aimed to evaluate the ability of social noncontact environmental enrich-ment to facilitate social buffering and to characterize the emotional experience of horses subjected to restraint in stock by assessing physiological parameters and facial expressions. We hypothesized that social enrichment would mitigate the short-term stress response, leading to a reduction in stress indicators associated with high arousal and negative valence.

## Materials and methods

### Ethics in animal research

This study was approved by the Animal Ethics Committee (CEUA) of the Federal University of Mato Grosso do Sul (UFMS, Campo Grande, Brazil) under protocol no. 1.222/2022.

### Animals and housing

Eleven Pantaneiro horses (4 geldings and 7 mares), ranging in age from 2.5 to 12 years (mean±SD: 6.0 ± 2.6 years) were selected to participate in this study. The horses had a mean body weight of 326.4 ± 23.7 kg, and their mean body condition score (BCS) was 5 ± 1 (according to the scale developed by Henneke *et al.* [28]). In addition, a 16-year-old Pantaneiro gelding, weighing 365.0 kg with a BCS of 6, was the conspecific assigned as a social companion in the SNE (Social Noncontact Enrichment) treatment. The selection criteria for this horse included the absence of kinship relationships with the evaluated animals, familiarity with them under routine farm conditions, no history of atypical aggression or frequent agonistic interactions, and recognition by the farm staff as a calm and cooperative individual during routine handling and management activities.

The animals belonged to the Federal University of Mato Grosso do Sul (UFMS) and were housed at the farm of the Faculty of Veterinary Medicine and Animal Science (FAMEZ), located in Terenos-MS (20° 26′ 18″ S, 54° 51′ 24″ W). The region’s climate is clas-sified as Aw (tropical savanna with dry winters) according to Köppen [29]. All selected individuals had been raised in social groups since birth and were housed in paddocks with *ad libitum* access to pasture (*Panicum maximum* cv. Tamani), mineral salt, and water. These housing conditions were maintained throughout the experimental period, except when the animals were undergoing evaluations.

### Experimental design

A crossover design was used, with treatments SNE and SI (Social Isolation during stock restraint). The animals were randomly assigned so that, in Period I, half of the animals received the SI treatment and the other half, the SNE treatment. In Period II, the treatments were alternated, allowing that, by the end, all animals had received both treatments. The interval between periods was one week, and treatments were administered once in each period for each animal, at the same time of day (7:30 am to 10:30 am).

### Habituation phase

Prior to the experimental phase, the animals underwent habituation to the location, equipment, and procedures required for data collection. During one week, the animals were led to the stock, where they remained for 3 minutes and were exposed to the equip-ment and procedures that would be carried out throughout the experiment. Horses were considered fit for participation when they met the following criteria: entering the stock without resistance and not displaying approach or avoidance behaviors toward the equipment during the procedures.

The animal assigned to serve as a companion for the SNE treatment was already fa-miliar with the location but also underwent habituation. However, instead of being re-strained in the stock, the animal was restrained by a halter in the location where it re-mained during data collection.

### Experimental phase

During the experimental phase, the animals were evaluated individually in the morning, with a total of four animals per day. In the SI treatment, each animal was led to the restraint stock (2.10 m × 0.88 m × 1.00 m), constructed with solid posts. The stock was located inside a covered barn area with partially enclosed lateral walls. These structural barriers prevented visual and physical contact with conspecifics. The remaining herd was maintained in a paddock located approximately 50 m from the evaluation site during data collection.

**Fig 1:**
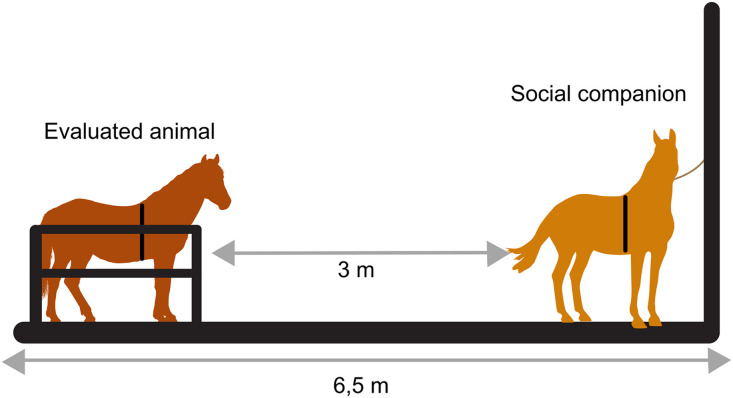
Experimental setup of the test area, as well as the location of the evaluated animal and the social companion during data collection (created by A.C.Bini de Lima using Canva resources).

For the SNE treatment, each animal was led to the same restraint stock used in the SI treatment. However, a familiar conspecific was positioned within the same covered barn area, outside the stock, at a distance of 3 meters, allowing visual, olfactory, and auditory communication (Fig 1). The partially enclosed lateral walls ensured that the restrained horse had visual access exclusively to the designated companion, preventing visual contact with other conspecifics and maintaining controlled experimental conditions. Since physical contact was not allowed, the social environmental enrichment tested in this study is characterized as social noncontact enrichment [7,8].

The experimental procedure was conducted similarly to that described by Fé *et al.* [27]. Each animal remained in the restraint stock for 24 minutes. This duration was defined to allow the extraction of three consecutive 5-minute intervals, which meet the minimum continuous recording time recommended for reliable heart rate variability (HRV) analysis [30] and enable longitudinal comparisons throughout the restraint period. Additionally, this duration allowed all four animals to be evaluated within the same morning period, minimizing environmental variation.

**Fig 2:**
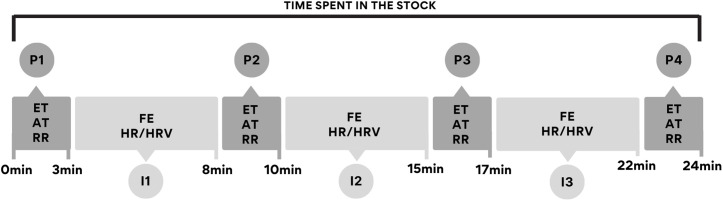
Experimental procedure. ET = eye temperature by infrared thermography; AT = auricular temperature by infrared thermometer; RR = respiratory rate; FE = facial expression; HR = heart rate; HRV = heart rate variability (created by **A.**C. Bini de Lima using Canva resources).

During the time spent in the stock, the parameters ET (eye temperature by infrared thermography), AT (auricular temperature by infrared thermometer), and RR (respiratory rate) were collected at specific time points P1, P2, P3, and P4. The parameters FE (facial expressions), HR (heart rate) and HRV were collected continuously but were analyzed only at the 5-minute intervals identified as I1, I2, and I3 (Fig 2).

### Environmental conditions

The study was carried out during the summer, between January and February 2022. In order to characterize the environment to which the animals were exposed and exclude thermal stress as a potential confounding factor, the following microclimatic parameters were monitored: dry bulb temperature (°C), black globe temperature (°C), dew point temperature (°C), wet bulb temperature (°C), relative humidity (%), and wind speed (m/s).

Measurements were taken at 10-minute intervals between 7:00 a.m. and 11:00 a.m. on collection days, with the aid of digital thermohygrometers (AK172®; AKSO, São Leopoldo, RS, Brazil), placed in meteorological shelters. For black globe temperature, the same model of thermohygrometer was used, encapsulated in PVC plastic spheres (15 cm in diameter) painted externally with matte black paint, as proposed by Souza *et al.* [31]. The equipment was placed in two locations: in direct sunlight and inside the facility where the animals remained during the tests, at a height of 1.50 m from the ground, during the solar exposure period, taking into account the variation in shadow projection and the zenith angle.

Based on the microclimatic data, the following were calculated: Radiant thermal load (W/m²), according to the equation proposed by Esmay [32]; Wet Bulb Globe Temperature (WBGT), as described by Schroter *et al.* [33]; Thermal Comfort Index (TCI), according to the equation proposed by Jones [34]. Using the obtained data, it was possible to characterize the environment and the thermal comfort condition of the animals during periods 1 and 2 (Table 1).

**Table 1:** Thermal environment and bioclimatic indices according to period.

Parameters	Period	Minimum	Maximum	Mean	Standard deviation
Air temperature (°C)	1	24	38	31	3.6
	2	23	34	28	2.7
Relative humidity (%)	1	48	87	67	10.9
	2	65	94	79	8.1
Radiant thermal load (W/m²)	1	452	643	532	50.6
	2	444	708	581	72.7
WBGT (°C)	1	22	34	28	3.4
	2	21	31	27	2.8
TCI	1	147	163	155	4.8
	2	154	170	161	5.0

Parameters: WBGT: Wet Bulb Globe Temperature; TCI: Thermal Comfort Index.

The air temperature, relative humidity, and radiant thermal load reflected what was expected for the region during this time of year, as the Aw climate is characterized by rainy summers with high temperatures [29]. Nevertheless, the average WBGT values ob-tained were ≤ 28 °C, indicating a low risk of heat stress [35,36].

The average values obtained for the TCI exceeded 130, which may indicate a thermally uncomfortable environment [34]. However, the horses used in this study belong to a locally adapted breed that has previously shown the ability to maintain relative homeostasis, even in conditions where the TCI exceeds 130 [27,37].

### Physiological parameters

#### Heart rate and heart rate variability.

The HR and HRV parameters were measured using the Polar H10 heart rate monitor (Polar Electro Oy, Kempele, Finland). The Polar H10 was attached to an elastic strap and positioned on the thoracic region, between the 4th and 5th intercostal spaces on the left side of the chest. The belt was moistened with water and adjusted to fit the animals’ bodies 10 minutes before data collection. To enhance the transmission of electrical signals from the body to the electrodes, the hair was cleaned with water.

The HR monitor and the video recordings for facial expression assessment were synchronized by starting them simultaneously, ensuring that video intervals directly corresponded to HR and HRV intervals. Data were collected and exported using the Elite HRV app (Elite HRV, Asheville, NC, USA), and analysis was performed using Kubios HRV Standard software, version 3.5.0 (Kubios Oy, Kuopio, Finland). A medium artifact correction was applied to minimize errors across the dataset, following the methodology of MCDUFFEE *et al.* [30], which allows for a correction of up to 15%. Among the 33 samples collected, only six had artifact values exceeding 5%, with the highest percentage of corrected artifacts in this study being 6.3%.

Five-minute intervals were selected for the analysis of HRV variables and mean heart rate. The following frequency domain HRV variables were chosen for this study: low-frequency power (LF, nu), high-frequency power (HF, nu), and the low-frequency to high-frequency ratio (LF/HF). The low-frequency (LF) band was defined as 0.04–0.15 Hz, while the high-frequency (HF) band was set at 0.15–0.4 Hz [27,30].

The HR of the conspecific assigned as the social companion in the SNE treatment was also monitored during the experiment to characterize its arousal during data collection (Table 2).

**Table 2:** Mean heart rate of the conspecific assigned to the SNE treatment as the companion during data collection.

Parameter	Period	Minimum	Maximum	Mean	Standard deviation
HR (bpm)	1	33.0	69.0	36.3	1.3
	2	31.0	50.0	34.6	1.5

The mean HR values recorded for the conspecific fell within the established reference range (28–40 bpm) for adult horses at rest, exposed to environments with temperatures between 22°C and 36°C [38,39]. The mean and standard deviation suggested that the animal exhibited predominantly low arousal during the observations.

#### Respiratory rate.

The RR, expressed in movements per minute (mpm), was measured by counting the flank movements over 30 seconds. The resulting values were then multiplied by two to determine the RR per minute.

#### Eye temperature by infrared thermography.

Infrared thermography images of the eye were captured to measure the surface tem-perature in the medial canthus region, as proposed by Kim and Cho [40]. The images were taken from the left side at an approximate 90° angle to the horses’ sagittal plane and a distance of 30–50 cm, in an area shielded from direct sunlight exposure.

The equipment used was a thermal camera (S60, Caterpillar FLIR camera, Vernon Hills, IL, USA) with a thermal resolution of 80 × 60 pixels, a visual resolution of 640 × 480, and a thermal sensitivity of 150 mK. The emissivity was set to 0.98, the standard value used for mammalian skin [41,42].

The FLIR Tools software, version 6.4.18039.1003 (FLIR Systems Inc., Oregon, USA), was used for the analysis of thermographic images. The distance at which the images were captured, as well as air temperature and relative humidity, were included in the software, which corrects for any environmental variations.

#### Auricular temperature by infrared thermometer.

The auricular temperature was measured using a portable digital thermometer with an infrared light device (Mult Temp Portátil, Incoterm, Porto Alegre, RS, Brazil), aimed at the central point of the auricular cavity on the left ear of the animal [27], at a distance of approximately 30 cm.

### Facial parameters

#### Video recording.

For the assessment of facial parameters, throughout the entire time the animal re-mained in the restraint stock, video recordings were made using a Canon EOS SL3 digital camera (Canon Inc., São Paulo, SP, Brazil). The resolution was set to 1080p at 30 fps, and the videos were exported in mp4 format. The camera was positioned 1.5 meters away at an approximate 45° angle to the medial plane of the horse [27].

#### Video processing and coding using the equine facial action coding system (EquiFACS).

The EquiFACS, as described by Wathan *et al.* [19], was used for coding facial expressions in all videos. For this study, 4 action units (AUs), 7 action descriptors (ADs), and one visibility code (VC) (Table 3) were selected, which correspond to facial expressions that have been shown to be relevant for assessing horses exposed to stressors in previous studies [21,27,43,44].

**Table 3:** Codes from EquiFACS selected for coding facial expressions in videos of horses subjected to short-term stress (stock restraint).

Code	Minimum criteria to code
I. Action Unit (AU)	
AU5: *Upper lid raiser*	An increase in the eye opening caused by the raising of the upper eyelid
AU145: *Blink*	Both eyelids must move together to cover the eye, and this action must be reversed within half a second
AU47: *Half blink*	A reduction in the opening of the eye
AU101: *Inner brow raiser*	Dorsal movement of the skin above the inner eye region
II. Action Descriptor (AD)	
AD1: *Eye white increase*	An increase in the percentage of white sclera visible
AD19: *Tongue show*	The tongue is shown and it reaches beyond the teeth.
AD38: *Nostril dilator*	An increase in the aperture of the nostril
AD81: *Chewing*	Minimum criteria to code not described
AD76: *Yawning*	Minimum criteria to code not described
EAD101: *Ears forward*	A rostral rotation of the pinna
EAD104: *Ear rotator*	The pinna rotates caudally
VC73: Entire face not visible	The entire face is either out of view or cannot be clearly seen

For coding facial expressions, 5-minute clips obtained during the time the animals spent in the restraint stock (I1, I2, and I3) were selected. All videos were coded by a single EquiFACS-certified coder, with inter-observer agreement >70% compared to experienced coders. For annotation, a list of the selected codes was entered into the BORIS software version 6.0.6 (Friard and Gamba, University of Turin, Turin, Italy). Continuous focal sampling was used to record the facial expressions of each horse. The clips were initially observed at normal speed and then observed at least three more times in slow motion or frame-by-frame to analyze each of the three regions of the horse’s face (ears, upper face, and lower face).

The frequency of each of the selected codes was recorded, except for VC73, for which the duration in seconds was recorded. VC73 was used to indicate how long the entire face was not visible for coding. This allowed for monitoring the duration in which the face was visible for coding during each 5-minute interval. When calculating the frequency per minute for the AUs and ADs, only the time during which the face was visible for coding was taken into account, according to the equation proposed by Fé *et al.* [27]*.* Out of 66 selected 5-minute clips, the face was in a visible position for coding in 61 for ≥ 4 minutes, in 4 for ≥ 3.5 minutes, and in only 1 for 2.75 minutes.

Intra-observer reliability was assessed using data obtained from five randomly se-lected 5-minute videos of horses participating in the study. In these videos, the frequency of each facial expression was recorded twice by the same observer on different occasions. To evaluate intra-observer reliability, the intraclass correlation coefficient (ICC) was cal-culated using a two-way model, with agreement definition and single measure statistic [45,46].

The ICC values range from −1–1 (complete disagreement to excellent agreement; ze-ro being agreement no different than chance) [47]. The ICC was 0.941 (95% CI: 0.900–0.965), indicating excellent reliability in the observer’s evaluations [45,48]. The F-test con-firmed that the agreement was significantly different from zero [F (54, 54.6) = 32.3, p < 0.001], reinforcing the robustness of the assessments. The analysis was performed using R software, with the *irr* package.

### Statistical analysis

All statistical analyses were performed in R software with the integrated develop-ment environment RStudio (Version 4.1.0 (2021-06-29), RStudio, Inc.). The functions and packages used are presented in the format ‘package::function’ corresponding to the R programming language. A significance level of 5% was considered for all tests.

Initially, an inferential analysis was performed to identify differences between treat-ments (SI vs SNE) and over time (P1 vs P2 vs P3 vs P4 or I1 vs I2 vs I3). Multilevel linear models (lme4::lmer) were conducted for the following response variables, for which the model residuals showed normality adherence according to the Cramer-Von Mises test (nortest::cvm.test): mean HR, LF, HF, LF/HF ratio, auricular temperature, inner *brow raiser* (AU101), *nostril dilator* (AD38), and *half blink* (AU47). The variables *ears forward* (EAD101) and *ear rotator* (EAD104) only met this condition after a Box-Cox transformation (car::powerTransform).

For discrete variables (respiratory rate), a generalized linear multilevel model was used, adjusted for the Poisson distribution. The interaction of time points or intervals with treatments, periods, and order were included as fixed effects in the model, while horses were included as random effects to account for individual variation. A multiple compari-son post-hoc test was conducted using the Bonferroni procedure (lsmeans::lsmeans and multcomp::cld) [49].

The variables eye temperature, *upper lid raiser* (AU5), *blink* (AU145), *eye white increase* (AD1), *tongue show* (AD19), *chewing* (AD81), and *yawning* (AD76) did not meet the normality assumption, even after transformations, preventing their modeling. Therefore, the comparison between treatments at the same time-point was performed using the paired two-tailed Wilcoxon paired Wilcoxon test (stats::wilcox.test), while the comparison over time for each treatment was conducted using the Friedman test (stats::friedman.test and PMCMRplus::frdAllPairsNemenyiTest).

The results were illustrated with boxplots (ggplot2::ggplot and ggplot2::geom_boxplot). Results for variables that met the normality assumption are presented in tables as mean and standard deviation (Mean ± SD), while results for variables that did not meet the normality assumption are presented as median and interquartile range (Median [IQR]).

Subsequently, an exploratory analysis was performed. To analyze the multivariate dynamics among the facial expressions assessed in this experiment, a principal compo-nent analysis (PCA) based on a correlation matrix (stats::princomp) was conducted. The optimal number of principal components (PCs) to retain in the PCA was determined us-ing Horn’s parallel analysis (‘psych::fa.parallel’) [50]. A loading value greater than 0.40 or less than −0.40 was used as the criterion to determine the association between each varia-ble and the PC [51]. To illustrate these results, two biplots were created, coloring the observations according to the treatments for a qualitative (visual) analysis of their distribution.

## Results

### Physiological parameters

The animals’ mean HR was significantly lower in the socially enriched environment compared to the socially isolated condition during the final 5-minute interval (I3) (P < 0.05). However, LF, HF, and LF/HF did not differ between treatments or across intervals (Table 4).

**Table 4:** Mean and standard deviation of cardiac parameters in horses during social isolation (SI) and social noncontact enrichment (SNE) treatments across three intervals.

Parameters	Treatments	Intervals		
		I1	I2	I3
Mean HR (bpm)	SI	39.3 ± 6.7	40.5 ± 7.4	**41.5 ± 8.9** ^**A**^
	SNE	38.3 ± 3.4	38.2 ± 3.5	**38.0 ± 3.9** ^**B**^
LF (nu)	SI	51.3 ± 10.3	61.3 ± 11.8	59.3 ± 15.2
	SNE	51.0 ± 15.7	51.5 ± 19.0	54.6 ± 19.7
HF (nu)	SI	48.7 ± 10.3	38.7 ± 11.8	40.6 ± 15.2
	SNE	49.0 ± 15.7	48.5 ± 19.0	45.4 ± 19.7
LF/HF	SI	1.1 ± 0.5	1.8 ± 0.9	1.8 ± 1.1
	SNE	1.2 ± 0.8	1.4 ± 0.9	1.6 ± 1.3

Parameters: HR: heart rate; LF: low-frequency power; HF: high-frequency power; LF/HF: low-frequency to high-frequency ratio. Different uppercase letters indicate statistically significant differences from the post-hoc test between treatments within the same interval (p < 0.05), with A > B.

**Fig 3:**
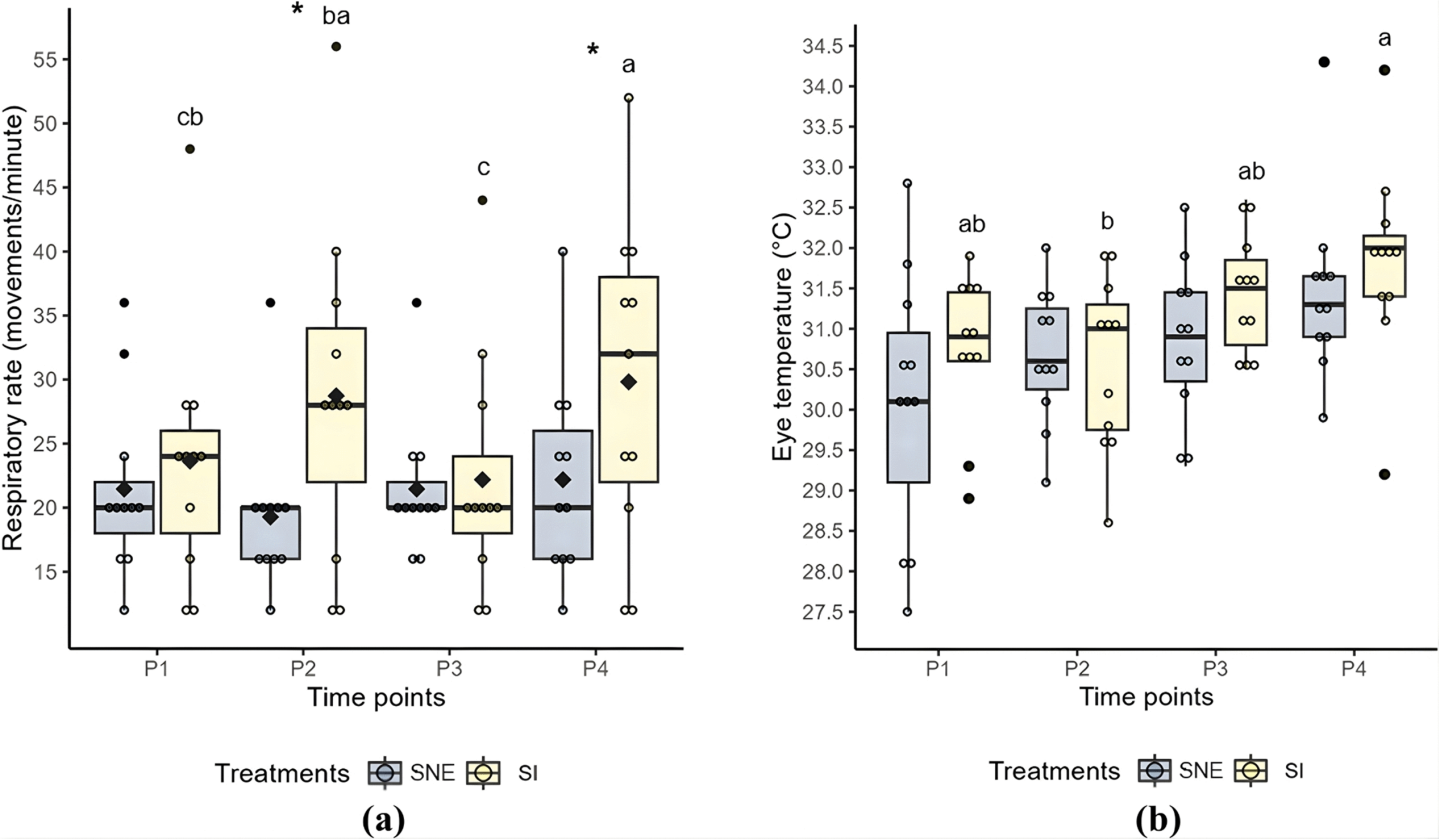
Boxplots: (a) Respiratory rate (movements/min); (b) Eye temperature (°C) during social isolation (SI) and social noncontact enrichment (SNE) treatments across four-time points. Different lowercase letters indicate statistically significant differences from the post-hoc test across time points for the same treatment (p < 0.05), with a > b; * indicates statistically significant differences from the post-hoc test between treatments at the same time point (p < 0.05); the black diamond represents the mean; each colored circle (yellow and blue) represents an individual animal, and each black circle indicates an outlier..

The respiratory rate in the SI treatment differed (P < 0.05) across time points. The highest values were observed at P2 and P4, where the respiratory rate was significantly higher for the SI treatment compared to the SNE treatment (P < 0.05) (Table 5; Fig 3).

**Table 5:** Mean and standard deviation or median and interquartile range of physiological parameters in horses during social isolation (SI) and social noncontact enrichment (SNE) treatments across the four-time points.

Parameters	Treatments	Time points			
		P1	P2	P3	P4
RR (mpm)	SI	**23.6 ± 9.9** ^**cb**^	**28.7 ± 13.0** ^**baA**^	**22.2 ± 9.4** ^**c**^	**29.8 ± 12.6** ^**aA**^
	SNE	**21.5 ± 7.0**	**19.3 ± 6.1** ^**B**^	**21.5 ± 5.5**	**22.2 ± 7.9** ^**B**^
AT (°C)	SI	31.9 ± 1.0	32.0 ± 1.0	32.3 ± 1.3	32.5 ± 1.2
	SNE	32.1 ± 0.9	32.4 ± 0.8	32.4 ± 0.8	32.3 ± 0.6
ET (°C)	SI	**30.9(0.9)** ^**ab**^	**31.0(1.6)** ^**b**^	**31.5(1.1)** ^**ab**^	**32.0(0.8)** ^**a**^
	SNE	**30.1(1.9)**	**30.6(1.0)**	**30.9(1.1)**	**31.3(0.8)**

Parameters: RR: respiratory rate; AT: auricular temperature by infrared thermometer; ET: eye temperature by infrared thermography. Different lowercase letters indicate a statistically significant difference from the post-hoc test across time points for the same treatment (p < 0.05), with a > b; different uppercase letters indicate a statistically significant difference from the post-hoc test between treatments within the same time point (p < 0.05), with A > B.

Auricular temperature was not influenced by the treatments or time points (Table 5). While no significant differences in eye temperature were observed between treatments, an increase in eye temperature was noted from time point P2 to P4 in the social isolation (SI) treatment (P < 0.05) (Table 5; Fig 3).

### Facial parameters

**Fig 4:**
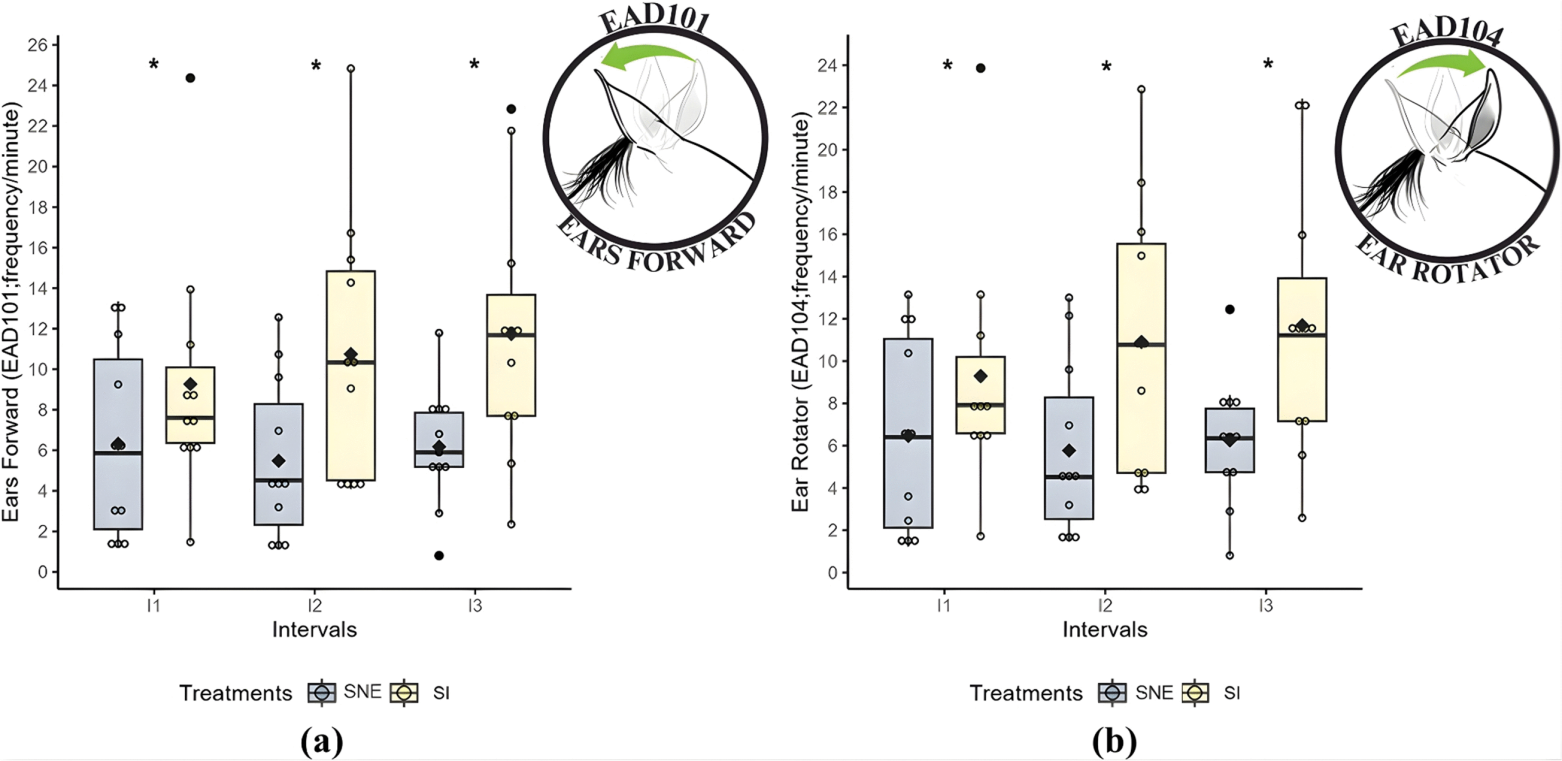
Boxplots: (a) *Ears forward* (EAD101; frequency/min); (b) *Ear rotator* (EAD104; frequency/min) during social isolation (SI) and social noncontact enrichment (SNE) treatments across three intervals. * Indicates a statistically significant difference in the post-hoc test between treatments at the same time point (p < 0.05); the black diamond represents the mean; each colored circle (yellow and blue) corresponds to an individual horse; and each black circle represents an outlier.

The frequency of the facial action descriptors *ears forward* (EAD101) and *ears backward* (EAD104) was lower during social noncontact enrichment compared to social isolation (P < 0.05) across all three intervals (Table 6; Fig 4).

**Table 6:** Mean and standard deviation or median and interquartile range of the frequency per minute of facial parameters expressed by horses during social isolation (SI) and social noncontact enrichment (SNE) treatments across three intervals.

Parameters	Treatments	Intervals		
		I1	I2	I3
*Ears forward* (EAD101)	SI	**9.3 ± 5.9** ^**A**^	**10.7 ± 6.6** ^**A**^	**12.0 ± 6.3** ^**A**^
	SNE	**6.3 ± 4.8** ^**B**^	**5.5 ± 4.0** ^**B**^	**6.2 ± 2.9** ^**B**^
*Ear rotator* (EAD104)	SI	**9.3 ± 5.6** ^**A**^	**10.9 ± 6.5** ^**A**^	**11.7 ± 6.3** ^**A**^
	SNE	**6.5 ± 4.7** ^**B**^	**5.8 ± 4.1** ^**B**^	**6.3 ± 3.1** ^**B**^
*Inner brow raiser* (AU101)	SI	**6.4 ± 3.0** ^**A**^	**6.3 ± 2.2** ^**A**^	**5.9 ± 2.3** ^**A**^
	SNE	**5.0 ± 2.6** ^**B**^	**4.2 ± 2.1** ^**B**^	**4.0 ± 3.0** ^**B**^
*Half blink* (AU47)	SI	11.0 ± 5.2	13.3 ± 6.6	12.8 ± 6.0
	SNE	10.3 ± 4.9	10.9 ± 4.7	10.0 ± 3.9
*Blink* (AU145)	SI	**13.9(3.9)**	**14.4(9.2)**	**12.1(8.5)**
	SNE	**16.0(6.5)** ^**ab**^	**16.4(7.1)** ^**a**^	**13.0(4.6)** ^**b**^
*Upper lid raiser* (AU5)	SI	**1.2(1.4)** ^**A**^	**1.7(1.7)** ^**A**^	**1.5(2.1)** ^**A**^
	SNE	**0.6(0.6)** ^**B**^	**0.6(0.9)** ^**B**^	**0.2(0.5)** ^**B**^
*Eye white increase* (AD1)	SI	**2.3(2.2)** ^**A**^	**1.7(2.3)** ^**A**^	1.4(2.3)
	SNE	**1.0(1.2)** ^**B**^	**0.5(1.7)** ^**B**^	0.4(1.1)
*Nostril dilator* (AD38)	SI	**19.9 ± 13.8** ^**A**^	**18.5 ± 10.8** ^**A**^	**21.7 ± 13.1** ^**A**^
	SNE	**11.9 ± 9.2** ^**B**^	**11.7 ± 7.4** ^**B**^	**10.2 ± 9.5** ^**B**^
*Yawning* (AD76)	SI	0.0(0.4)	0.3(0.4)	0.0(0.2)
	SNE	0.4(0.8)	0.0(0.4)	0.0(0.1)
*Chewing* (AD81)	SI	0.9(1.8)	1.2(1.7)	0.5(0.6)
	SNE	1.2(1.3)	0.6(1.3)	0.2(0.6)
*Tongue show* (AD19)	SI	0.7(5.5)	1.9(3.2)	1.2(2.9)
	SNE	2.0(3.4)	2.7(4.5)	0.4(1.6)

Lowercase letters indicate a statistically significant difference in the post-hoc test across intervals within the same treatment (p < 0.05), with a > b. Uppercase letters indicate a statistically significant difference in the post-hoc test between treatments within the same interval (p < 0.05), with A > B.

**Fig 5:**
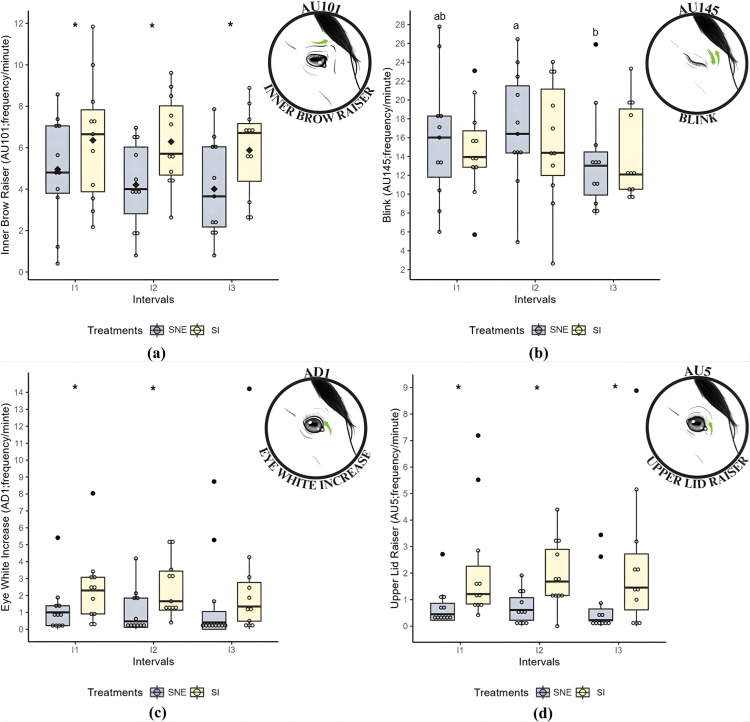
Boxplots: (a) *Inner brow raiser* (AU101; frequency/min); (b) *Blink* (AU145; frequency/min); (c) *Upper lid raiser* (AU5; frequency/min); (d) *Eye white increase* (AD1; frequency/min) during social isolation (SI) and social noncontact enrichment (SNE) treatments across three intervals. Different lowercase letters indicate statistically significant differences from the post-hoc test across intervals for the same treatment (p < 0.05), where a > b; * indicates statistically significant differences from the post-hoc test between treatments at the same interval (p < 0.05); the black diamond represents the mean; each colored circle (yellow and blue) represents an individual animal; and each black circle indicates an outlier.

The frequency of *inner brow raiser* (AU101) was lower in the SNE treatment compared to SI (P < 0.05) in all three intervals (Table 6; Fig 5[a]). For *blinking* (AU145), a difference (P < 0.05) was observed across the intervals for SNE, with the highest frequency recorded in I2 and the lowest in I3 (Table 6; Fig 5[b]). Meanwhile, the frequency of *eyelid raiser* (AU5) was lower in the SNE treatment compared to SI (P < 0.05) in all intervals (Table 6; Fig 5[c]). For *eye white increase* (AD1), the frequency was lower in the SNE treatment compared to SI (P < 0.05), but only in intervals I1 and I2 (Table 6; Fig 5[d]).

**Fig 6:**
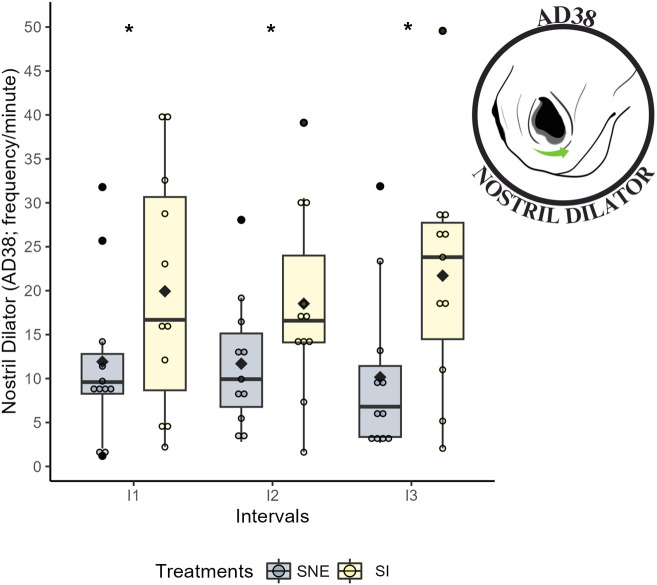
Boxplot: *nostril dilation* (AD38; frequency/min) during the social isolation (SI) and social noncontact enrichment (SNE) treatments across three intervals. * Indicates a statistically significant difference from the post-hoc test between treatments at the same time (p < 0.05); the black diamond represents the mean; each colored circle with treatment colors (yellow and blue) represents an animal; and each black circle indicates an outlier.

The facial action descriptor *nostril dilation* (AD38) was observed at a lower frequen-cy for the SNE treatment compared to the SI treatment (P < 0.05) in all intervals (Table 6; Fig 6).

The Horn’s parallel analysis indicated the retention of the first and second PCs out of a total of 5 PCs generated by the PCA, so only these were analyzed. The PCA loadings indicate the level of association between a variable and a given PC, with load values further from zero indicating a higher level of positive (1.00) or negative (−1.00) association. Therefore, the set of variables positively associated with a PC exhibit a similar dynamic among themselves, as all increase or decrease concomitantly.

The associations were determined by a cutoff of 0.40 for both positive and negative load values. PC1 alone explained almost half (42.2%) of the total variance of the data, with *ears forward* (EAD101), *ears rotator* (EAD104), *inner brow raiser* (AU101), *half blink* (AU47), *upper lid raiser* (AU5), *eye white increase* e (AD1), *nostril dilation* (AD38) showing positive associa-tions (load values >0.40) with PC1 (Table 7). Thus, the decrease or increase of these facial expressions occurred concurrently across treatments. *Ears rotator* (EAD104) showed the load value further from zero on PC1, which can be interpreted as the facial expression with the greatest variation (importance) on PC1.

**Table 7:** Loading values, eigenvalues, and variance from the principal component analysis (PC = principal component; values in bold indicate loadings greater than 0.40 or less than −0.40, indicating the variable’s association with the PC).

Parameters	PC1	PC2
Ears forward (EAD101)	**0.809**	−0.134
Ear rotator (EAD104)	**0.814**	−0.127
Inner brow raiser (AU101)	**0.745**	−0.109
Half blink (AU47)	**0.613**	−0.124
Blink (AU145)	0.070	**0.417**
Upper lid raiser (AU5)	**0.757**	−0.390
Eye white increase (AD1)	**0.742**	−0.349
Nostril dilator (AD38)	**0.594**	−0.386
Yawning (AD76)	**0.442**	**0.753**
Chewing (AD81)	**0.648**	**0.664**
Tongue show (AD19)	**0.544**	**0.663**
Eigenvalues	4.641	2.104
Variance	42.187	19.123
Cumulative variance	42.187	61.311

*Yawning* (AD76), *chewing* (AD81), and *tongue show* (AD19) demonstrated positive as-sociations (load values >0.40) with both PC1 and PC2. *Blink* (AU145) was the only facial expression that demonstrated a positive association only with PC2 (Table 7).

**Fig 7:**
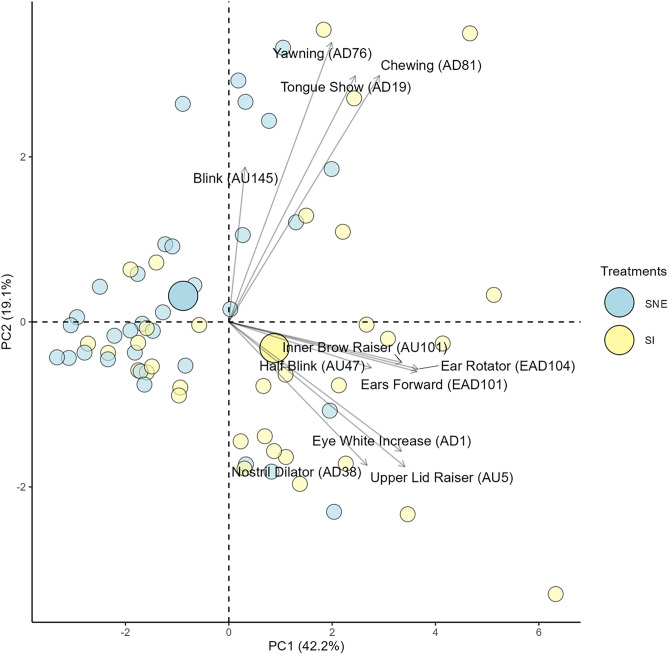
Two-dimensional biplot of the principal component analysis with the facial expressions and observations separated by treatment (Smaller circles indicate each evaluation and larger cir-cles represent the centroid of each treatment; the centroid indicates the center of mass given by the polygon formed by the interpolation of the smaller circles of the same color; the arrows indicate the vectors of each facial expression).

Fig 7 separates the evaluations of the horses in the social isolation (SI) treatment and the social noncontact enrichment (SNE) treatment using different colors. One of the pieces of information in this figure is the centroid (larger circle), which indicates the center of mass given by the polygon (geometric shape) formed by the interpolation of the evaluations (smaller circles) of the same color. Based on a visual judgment, the centroids of both treatments are separated, indicating that the facial expressions were able to distinguish the treatments.

Moreover, the centroid of the social isolation treatment is located in the lower-right quadrant, where the vectors of *ears forward* (EAD101), *ear rotator* (EAD104), *inner* *brow raiser* (AU101), *half blink* (AU47), *upper lid raiser* (AU5), *eye white increase* (AD1), and *nostril dilation* (AD38) are found, suggesting that these facial expressions are more associated with the social isolation treatment. These results explore the potential multiple dynamics of the facial expressions, which were able to distinguish treatments in the previous inferential analyses.

## Discussion

This study investigated whether the use of social environmental enrichment without physical contact could facilitate social buffering, reducing physiological responses and facial expressions associated with short-term stress in horses. As this is a potentially aversive and stressful intervention frequently performed in equestrian centers, restraint in stock was chosen for this study, increasing its relevance to the welfare of domestic horses.

In general, the physiological stress response is characterized by increased heart and respiratory rates due to the activation of the sympathetic branch of the autonomic nervous system [17,52,53]. The mean heart rate values recorded in both treatments remain within or close to the reference range (28–40 bpm) established for adult horses at rest under envi-ronmental conditions similar to those of the present study (22°C–36°C) [38,39]. However, the observed cardiac responses remain consistent with previous studies in which horses were exposed to stressful events, including movement restriction [27,54–56].

The highest heart rate recorded in this study (41.5 bpm), observed in the last 5-minute interval (I3) when the horses were under movement restriction and social isolation, was similar to the value reported by Fenner *et al.* [56] (46.28 bpm) when horses were subjected to movement restriction and the use of restrictive equipment (tight noseband) capable of inducing a physiological stress response and inhibiting the expression of oral behaviors. Likewise, Yarnell *et al.* [54] reported heart rates near 40 bpm in cooperative horses exposed to an aversive procedure involving movement restriction and unpleasant auditory stimulus (clipping). Our previous study on auditory enrichment [27] also reported heart rates mostly in the 40s in Pantaneiro horses under conditions similar to the present study, further supporting the consistency of these physiological responses.

The subtle and gradual increase in heart rate that resulted in the peak observed in I3 for the SI treatment can be explained by the horses remaining stationary during the tests. The lack of physical activity likely contributed to heart rate values closer to the reference range typically obtained in resting animals [38,39]. The nature of the stressor should also be considered, as more pronounced responses generally occur when stressors are pre-sented abruptly [6,57], which was not the case in this study. Another contributing factor may be the habituation of the animals to the procedure, as this process typically leads to a reduction in response to the stimulus [58,59]. All animals were cooperative during the tests, and none showed resistance when entering or leaving the restraint stock.

In the same interval (I3), the heart rate was lower when the company of a conspecific was provided as social enrichment (SNE). This response is consistent with evidence that social support can have a positive impact on the cardiovascular system, reducing heart rate during stressful tasks in various species [4]. Lower heart rates have been reported in horses exposed to a frightening stimulus [60] or subjected to transport [55] when accom-panied by a conspecific.

For respiratory rate, animals treated with social enrichment maintained stable mean values within the reference range (10–20 breaths per minute, [38,39]) throughout the observation period. In contrast, when isolated, they exhibited an inconsistent respiratory rate, with fluctuations over time and values further from the reference range. The highest value was recorded when the duration of restraint approached 20 minutes. At the same time, the highest heart rate was observed. These results indicate that the restraint proce-dure induced physiological stress responses, which were mitigated when animals were provided with the company of a conspecific, suggesting the occurrence of social buffering.

Changes in eye temperature have been associated with aversive and/or negative pro-cedures and are recognized as a stress response in mammals, caused by sympathetically mediated alterations in blood flow [61,62]. Significant fluctuations in eye temperature were observed over time when the animals were isolated during stock restraint (SI). In contrast, when accompanied (SNE), the animals’ eye temperature exhibited minimal vari-ation throughout the time spent in the stock.

The increase observed in eye temperature (1.0 °C from P2 to P4 [SI]) is consistent with findings from other studies involving stressful events, such as jugular catheterization in dairy cows (0.9 °C) [63] and the use of restrictive equipment (tight noseband) in horses under movement restriction (0.4 °C) [56]. The rise in eye temperature during the SI treat-ment peaked alongside increases in heart and respiratory rates, suggesting that, over time, the physiological stress responses were exacerbated. Duration of exposure may play a key role in intensifying the effects of this type of stressor, as demonstrated in previous research. For instance, Vollenhoven *et al.* [3] reported that the stress response in mares was more pronounced after prolonged movement restriction in stocks, and our previous study on auditory enrichment [27] similarly showed a progressive rise in eye temperature in Pantaneiro geldings, with the peak occurring approximately 17 minutes after the onset of restraint.

This pattern of increased eye temperature in response to a stressor is likely driven by the dilation of ocular blood vessels and heightened visual attention/orientation [54]. Consistent with this, in the present study, eye-related facial expressions that expand the visual field were more frequently observed in isolated horses. *Upper lid raiser* (AU5), which increases eye-opening by elevating the upper eyelid, can lead to greater scleral exposure, identified as *eye white increase* (AD1) [19]. In horses, both *upper lid raiser* (AU5) and *eye white increase* (AD1) have been linked to stress during transportation and social isolation [21]. Furthermore, *eye white increase* (AD1) has been correlated with attention orientation in this species [64].

Increased scleral exposure has been suggested to be more closely associated with heightened arousal levels rather than emotional valence, as this expression has been iden-tified in contexts related to both positive and negative high-arousal emotional states in horses [25] and cattle [65]. Furthermore, it is suggested that a rise in the expression of *eye white increase* (AD1) could signal intense arousal, as sympathetic axons innervate the muscle responsible for elevating the upper eyelid [64,66].

The action unit *inner brow raiser* (AU101) was also expressed more frequently by horses when isolated. This expression is characterized by a dorsal movement of the skin above the inner eye region [19], giving the eye a triangular shape, which is generally em-pirically associated with pain and has even been included as a facial parameter in some tools developed for pain assessment in horses [67,68]. However, with the development of EquiFACS, it became possible to reliably code facial movements from video sequences. Studies using this system have shown inconsistent results for the *inner brow raiser* (AU101) [69] or demonstrated that this AU was unable to predict pain [70].

Using EquiFACS in the context of potentially stressful situations (transportation and social isolation), the *inner brow raiser* (AU101) was identified as a relevant facial action unit [21]. Similar to Lundblad *et al.* [21], the present study recruited animals perceived as healthy and used them as their own control, thereby minimizing the risk of the presence of pain. A distinguishing feature of this study was the coding of longer video sequences. Thus, the results reinforce the significance of this facial expression for identifying stress in horses subjected to social isolation and demonstrate that social enrichment can influence the expression of this AU.

In contrast to the other eye-related expressions, which were more frequently exhibited when the horses were isolated during stock restraint, *blink* (AU145) displayed a distinct pattern, varying over time only when the horses were in the company of a conspecific. Spontaneous blink rate has been used as an indicator of stress in horses [21,43,71,72], but the results reported thus far have been inconsistent, with both increases [21] and decreases in blink rate observed in socially isolated horses [43].

In the present study, no differences in the expression of this facial action unit were noted when the horses were isolated, consistent with our previous findings [27], in which the horses were also socially isolated and restrained in a stock. Moreover, the pattern observed when they received social enrichment, showing an increase in frequency during I2 followed by a decrease in I3, was the opposite of what was described by Mott *et al.* [72], where horses exposed to a stressful event exhibited a decrease followed by an increase in blink rate.

The blink pattern observed in this study may be associated with the neurobiological mechanisms underlying social buffering, which, although not yet fully elucidated, suggest that social interactions lead to oxytocin release in the amygdala and a subsequent positive effect on dopamine transmission in the nucleus accumbens [5,73], considered the primary structure of the ventral striatum. The spontaneous blink rate is mediated by the activation of the D1 and D2 dopamine receptors in the striatum [72]. Therefore, an in-crease in dopamine in this brain region during the presentation of rewards or positive events, such as social interactions, could lead to an increase in this facial expression. While these mechanisms partially explain the observed results, additional research is needed, especially studies that explore the link between this facial action unit and social buffering.

Horses isolated during restraint exhibited greater nostril dilation compared to when they received social enrichment. Nostril aperture can change in diameter depending on the animal’s physiological and psychological state, with dilated nostrils generally associ-ated with an alert posture [74], attention and avoidance [75], and pain [69,70]. This facial expression is linked to olfactory investigation, allowing the horse to gather information about its environment when in a state of alertness and attention [74,76], as well as being associated with the increased respiratory rate that occurs during stressful events due to sympathetic activation, preparing the body for a possible fight-or-flight response [53,59]. Not surprisingly, previous findings have associated *nostril dilation* (AD38) with stressful interventions such as transportation, social isolation and stock restraint [21,27,77] in ex-periments involving horses. Thus, the results observed here align with previous findings and are supported by the physiological responses discussed earlier, as the highest rec-orded values for respiratory rate were also observed in the animals assessed during isola-tion.

The position and movement pattern of the ears have been associated with emotional states in animals with mobile ears, such as horses [22,75,78]. Ear movement differed be-tween treatments, with horses in isolation moving their ears forward and backward more frequently. This movement pattern aligns with that described by Lundblad *et al.* [21], who reported an increase in the “ear flicker” movement index, used to describe whether *ears forward* (EAD101) and *ear rotator* (EAD104) occurred together within one second during stressful events (transportation and social isolation). A likely cause of these move-ments would be the increased perception of surroundings due to agitation and/or alert-ness induced by social isolation.

This ear movement pattern, characterized by the facial action descriptors *ears* *forward* (EAD101) and *ear rotator* (EAD104), may correspond to the “asymmetric ears” described in the literature, a behavioral pattern often associated with negative emotional experiences [75,78]. It has been shown that asymmetric ears may reflect negative emotions in horses groomed with a fixed procedure known to induce avoidance reactions [79]. In the study by Lansade *et al.* [79], asymmetric ears were more frequently observed alongside expressions such as eyes wide open and scleral exposure. Therefore, to elucidate how ear position and movement may be associated with emotional state, it is important to observe other facial expressions in conjunction.

To analyze the multiple dynamics between the facial expressions assessed in this study, a principal component analysis was conducted. A positive association with PC1 was observed for the expressions *ears forward* (EAD101), *ear rotator* (EAD104), *inner brow raiser* (AU101), *half blink* (AU47), *upper lid raiser* (AU5), *eye white increase* (AD1), *nostril dilator* (AD38), showing that these expressions exhibited a similar dynamic, increasing or decreasing simultaneously across the treatments. Furthermore, the PCA results corroborated the findings from the previous inferential analyses, suggesting that these facial expressions are more associated with the social isolation treatment.

In contrast, the centroid of the social noncontact enrichment treatment, positioned in the positive quadrant of PC2, suggests a weaker association between this treatment and stress-related facial expressions. *Blink* (AU145) was the only facial expression that showed a positive association exclusively with PC2, demonstrating that this expression behaved antagonistically to the other expressions considered relevant in stressful contexts. This may imply that this action unit is relevant to the study of social buffering in horses.

The facial expressions associated with oral movements, such as *yawning* (AD76), *chewing* (AD81), and *tongue show* (AD19), although not differing between treatments in the inferential analysis, exhibited a positive association with both PC1 and PC2 in the PCA, showing a similar dynamic among them. This pattern aligns with previous reports suggesting that yawning can occur intermittently with chewing and tongue exposure [44]. Furthermore, these oral movements are considered part of a cluster of autonomic re-sponses that follow an acute sympathetic surge [44], resulting from the subsequent de-crease in sympathetic activity and the activation of the parasympathetic nervous system [59]. This may explain the positive association with both principal components. Under-standing this aspect may help clarify the distinct characteristics of facial expressions used as stress indicators. It is plausible that some expressions occur during the peak of sympa-thetic activity, while others occur afterward. This distinction could be relevant for identi-fying the most suitable expressions to use, depending on the context and type of stressor. In this study, the stressor the animals were exposed to showed a gradual effect, and the peak of sympathetic activity appears to have occurred during the final 5-minute interval. This may explain the lack of significance for these facial parameters, as no data were col-lected after the peak.

This study demonstrates that the movement restriction imposed by restraint in a stock can trigger a stress response, even in habituated animals, especially when they are isolated. When horses were subjected to social isolation during restraint, significant changes in physiological parameters were observed, including an increase in heart rate, respiratory rate, and eye temperature. These findings confirm that the intervention dis-rupted the sympathetic-vagal balance and heightened arousal. Additionally, a higher frequency of facial expressions linked to stress responses was observed, such as *nostril dilator* (AD38), *inner brow raiser* (AU101), *upper lid raiser* (AU5), *eye white increase* (AD1), *ears forward* (EAD101), and *ears rotator* (EAD104). These expressions are commonly associated with both increased arousal and negative valence emotional experiences.

Collectively, the physiological and facial parameters assessed in this study suggest that the strategic use of a conspecific as social noncontact enrichment during stock re-straint can facilitate social buffering, characterized by the mitigation of stress responses. Several key factors may have contributed to these findings, which align with previous re-search. These include the presence of a calm social companion [60] and the opportunity for socialization through visual, auditory, and olfactory communication, as sensory mo-dalities essential for social interactions among conspecifics are generally important for mediating social buffering [52,75,76,80].

Although stock restraint may be perceived as a negative experience, it is an integral part of stressful procedures associated with proper husbandry practices. In this context, social enrichment offers animals the opportunity to effectively cope with this stressor, leading to positive impacts on their welfare. Additionally, social enrichment can improve handling efficiency and enhance safety, as calmer animals are more cooperative and easi-er to manage.

## Conclusions

Stock restraint induces a stress response even in habituated animals, especially when they are isolated. The assessment of facial expressions alongside physiological parame-ters provides robust evidence that, during this intervention, animals experience an emotional state characterized by high arousal and negative valence. In this context, the strategic use of a conspecific as social noncontact enrichment can facilitate social buffering, leading to a reduction in stress indicators associated with high arousal and negative valence.
